# Standardized amino acid digestibility and true metabolizable energy for several increased protein ethanol co-products produced using back-end fractionation systems

**DOI:** 10.1016/j.psj.2022.102329

**Published:** 2022-11-10

**Authors:** B.W. Parsons, P.L. Utterback, C.M. Parsons, J.L. Emmert

**Affiliations:** Department of Animal Sciences, University of Illinois at Urbana-Champaign, IL 61801, USA

**Keywords:** amino acid digestibility, true metabolizable energy, distillers dried grains with solubles, corn fermented protein

## Abstract

Precision-fed rooster trials were conducted to evaluate standardized AA digestibility and TME_n_ of the increased protein ethanol co-products corn fermented protein (**CFP**), high protein-distillers dried grains with solubles (**HP-DDGS**), and reduced fiber high protein-DDGS (**RFHP-DDGS**) produced using post-fermentation back-end fractionation systems. The TME_n_ was determined using conventional adult Leghorn roosters, while cecectomized roosters were used to determine standardized AA digestibility. Three to 6 roosters were fasted per treatment for 26 h prior to crop intubation with 27 g of sample and excreta were collected for 48 h post-feeding. Statistical analyses were conducted using a one-way ANOVA for a completely randomized design. Eight samples of CFP were found to contain a mean of 56% CP (DM basis) compared with a mean of 32% for conventional DDGS. The mean TME_n_ of CFP (3,556 kcal/kg) was greater (*P* < 0.05) than conventional DDGS1 and 2 (2,767 kcal/kg DM); mean standardized AA digestibility for CFP was similar to conventional DDGS and ranged from 88 to 94%. The mean digestible Lys, Met+Cys, and Thr concentrations for conventional DDGS were 0.79, 1.12, and 0.94%, respectively, whereas those for CFP were 1.74, 2.06, and 1.88%, respectively (DM basis). Two samples of HP-DDGS contained a mean of 51% CP (DM basis), a mean TME_n_ of 3,325 kcal/kg DM, a mean standardized AA digestibility of 90%, and mean concentrations of digestible Lys, Met+Cys, and Thr, which were 1.53, 1.77, and 1.60%, respectively (DM basis). The mean CP content of 2 RFHP-DDGS was 48% and the mean TME_n_ was 3,711 kcal/kg DM, which was greater (*P* < 0.05) than conventional DDGS3 and 4 (2,920 kcal/kg DM). Mean standardized AA digestibility of RFHP-DDGS was 90% and mean digestible Lys, Met+Cys, and Thr concentrations increased from 0.82, 1.01, and 0.95% for conventional DDGS, respectively, to 1.00, 1.59, and 1.44% for RFHP-DDGS, respectively (DM basis). Results indicate these high protein corn ethanol co-products have increased nutritional value for poultry compared with conventional DDGS.

## INTRODUCTION

Distillers dried grains with solubles (**DDGS**) is one of the primary co-products from the fuel ethanol industry. Ethanol production from dry grind processing of corn has increased rapidly over the past 2 decades, which has subsequently increased the supply of corn DDGS (RFA, 2022). The expansion of ethanol production has begun to slow in recent years and demand is expected to decrease over time due to increased use of electric vehicles (AFDC, 2022). Projected reductions in demand for fuel ethanol combined with increased fluctuation in ethanol prices have resulted in ethanol producers having increased interest in developing revenue streams other than ethanol and DDGS. Distillers dried grains with solubles is used primarily in ruminant feed due to its high fiber content and high degree of variability (Singh et al., 2005); however, recent economic pressures combined with increased supply of DDGS have provided more incentive for ethanol plants to produce higher quality DDGS that is more marketable to monogastric animal industries.

The poultry industry currently utilizes approximately 7% of the DDGS produced in the United States (RFA, 2022). Conventional corn DDGS contains approximately 27.4% CP on an as-fed basis and is low in both total Lys and Trp (NRC, 1994). On a DM basis, DDGS also contains 41.9% neutral detergent fiber (**NDF**), which has limited digestibility in monogastric animals (Urriola et al., 2010). In order to produce DDGS that has increased nutritional value for monogastric animals, modified front-end processing systems, in which fractionation occurs prior to fermentation, were initially developed, and several have been evaluated (Singh et al., 2005; Martinez Amezcua et al., 2007; Kim et al., 2010). More recently, there has been increased emphasis on developing modified back-end processing systems, where fractionation occurs after fermentation, to increase CP and decrease fiber content of DDGS. For example, Corray et al. (2019) reported that the back-end system developed by Fluid Quip Technologies (Cedar Rapids, IA) produced a DDGS that contained approximately 50% CP on an as-fed basis. This system produces a high protein product now called mechanically separated corn fermented protein (**CFP**), by utilizing mechanical fractionation in which whole stillage is subjected to fiber washing, protein filtration, protein washing, and protein drying. Currently, 7 plants in the United States are producing an average quantity of 500,000 tons of CFP annually, with a projected annual production of approximately 1,000,000 tons by the end of 2022, P. Williams (Fluid Quip Technologies, personal communication). The CFP is produced from a system similar to the system used to produce the one sample of the Still Pro product evaluated by Corray et al. (2019), which contained 53% CP, 5.1% fat, and 24.1% NDF on a DM basis, and digestible amino acid (**AA**) concentrations that were higher than conventional DDGS (NRC, 1994).

Other examples of novel increased protein co-products are high protein-DDGS (**HP-DDGS**), produced by Franzenburg (Des Moines, IA), and a reduced fiber high protein-DDGS (**RFHP-DDGS**), produced from removal of fiber by ST Equipment and Technology LLC (Needham, MA). These products have not been evaluated previously. Increased development and implementation of new back-end processing systems has led to a greater need for nutritional evaluation of the high protein corn co-products produced from these systems so they can be effectively utilized in poultry diets.

The precision-fed rooster assay is frequently used for nutritional evaluation of feed ingredients. The assay was first developed by Sibbald (1976) for TME and has since been modified to measure both TME and AA digestibility (Engster et al., 1985). In this assay, conventional Single Comb White Leghorn roosters are used for the evaluation of TME_n_, whereas cecectomized roosters are used for evaluation of standardized AA digestibility (Parsons, 1985). This assay has frequently been used for evaluation of DDGS and modified co-products from ethanol production (Batal and Dale, 2006; Martinez Amezcua et al., 2007; Kim et al., 2008; Pahm et al., 2009; Kim et al., 2010; Corray et al., 2019). The objective of this study was to determine the standardized AA digestibility and TME_n_ of several novel increased protein ethanol co-products from dry grind processing of corn for fuel ethanol production, produced using back-end fractionation systems.

## MATERIALS AND METHODS

The protocol for this study was reviewed and approved by the Institutional Animal Care and Use Committee at the University of Illinois (protocol number 20131).

### Ingredients and Analyses

A total of 17 samples of corn co-products obtained from modified back-end fractionation systems and conventional corn DDGS were evaluated in this study. Eight samples of CFP were obtained from various ethanol plants that use equipment provided by Fluid Quip Technologies and 2 samples of conventional DDGS were also obtained. The CFP samples were collected over a period of 3 yr, from 2017 to 2020. Two samples of HP-DDGS, produced from a different back-end fractionation system that uses sieving, were provided by Franzenburg. Lastly, 2 samples of RFHP-DDGS, 1 sample of a low protein DDGS (**LP-DDGS**), and 2 samples of conventional DDGS were obtained from ST Equipment and Technology LLC. The RFHP-DDGS was produced using a triboelectrostatic belt separator, which separates fiber from protein by utilizing differences in charges of particles. The LP-DDGS was the remaining fraction left after the recovery of the RFHP-DDGS. Analyses were conducted by the Agricultural Experiment Station Chemical Laboratory (University of Missouri, Columbia, MO) to determine CP by measuring nitrogen content via combustion (Method 990.03; AOAC International, 2007), crude fat (Method 920.39 A; AOAC International, 2007), NDF (Method 2002.04; AOAC International, 2007), ash (Method 942.05; AOAC International, 2007), Ca, P, and Na via inductively coupled plasma optical emission spectrometry (Method 958.01 A, B, and D; AOAC International, 2007), and AA concentrations (Method 982.30 E [a, b, and c]; AOAC International, 2007). Samples were analyzed for dry matter content using method 930.15 (AOAC International, 2007) and insoluble dietary fiber (**IDF**) and soluble dietary fiber (**SDF**) using method 991.43 (AOAC International, 2007) at the University of Illinois. The IDF and SDF were determined using an Ankom Total Dietary Fiber Analyzer (Ankom Technology, Macedon NY) and total dietary fiber (**TDF**) was calculated using the sum of IDF and SDF. Gross energy analyses were performed by NP Analytical Laboratories (St. Louis, MO).

### Diets and Experimental Design

Conventional and high protein co-products from dry grind ethanol plants were evaluated for TME_n_ and standardized AA digestibility. Experiments were conducted over a 2-yr period with Single Comb White Leghorn roosters (5 experiments with conventional roosters and 5 experiments with cecectomized roosters) using the precision-fed rooster assay (Sibbald, 1976; Parsons et al., 1982; Parsons, 1985). Four experiments were conducted to determine TME_n_ and standardized AA digestibility of CFP samples, 2 experiments were conducted to evaluate HP-DDGS samples, and 4 experiments were conducted to evaluate RFHP-DDGS, LP-DDGS, and conventional DDGS obtained from ST Equipment and Technology LLC. The TME_n_ was determined in conventional roosters and AA digestibility was determined in cecectomized roosters. The cecectomies were performed as described by Parsons (1985). Roosters were fasted for 26 h prior to being precision-fed (crop intubated with) 27 g of sample. The CFP samples obtained from Fluid Quip Technologies and the LP-DDGS and RFHP-DDGS from ST Equipment and Technology were fed as a mixture (50% corn) because of the powdery texture and small particle size. The conventional DDGS samples were also fed as a 50% mixture with corn to be consistent with the method in which the CFP, LP-DDGS, and RFHP-DDGS were fed. The HP-DDGS samples obtained from Franzenburg were fed as a single ingredient, not as a mixture. An additional 8 roosters were also precision-fed 30 g of corn. The same sample of corn (stored frozen) was fed in all rooster assays. All samples were fed in mash form. There were 3 to 6 replicates of one individually caged rooster per treatment and the age of the roosters varied from 40 to 60 wk among assays. The number of replicates varied among treatments because of variation in the amount of sample and roosters that were available. All birds were provided water ad libitum. Excreta were quantitatively collected for 48 h post-feeding on trays placed underneath each cage, after which excreta were stored in a freezer prior to lyophilization. After lyophilization, dried excreta were weighed and ground. Excreta from conventional roosters were analyzed for gross energy and nitrogen, whereas excreta from cecectomized roosters were analyzed for AA as described previously.

The TME_n_ of the diets was calculated as described by Parsons et al. (1982) and the TME_n_ values of test ingredients were calculated by difference using the method of Han et al. (1976) when the test ingredients were fed as a mixture with corn. Basal endogenous energy losses were determined from gross energy and nitrogen analysis of excreta collected from conventional roosters that were fasted for 48 h instead of being precision-fed. The equations are shown below:

TME_n_ of diets (kcal/g) =
[gross energy consumed – (gross energy excreted by fed birds + 8.22 × nitrogen retained by fed birds) + (gross energy excreted by fasted birds + 8.22 × nitrogen retained by fasted birds)] / feed intake (g)

where gross energy consumed (kcal) = diet intake (g) × gross energy of the diet (kcal/g); gross energy excreted by fed or fasted birds (kcal) = excreta output (g) × gross energy of excreta (kcal/g); 8.22 = gross energy (kcal) of uric acid per g of nitrogen (Hill and Anderson, 1958); nitrogen retained by fed or fasted birds (g) = diet intake (g) × diet nitrogen (%) - excreta output (g) × excreta nitrogen (%).

The TME_n_ values of CFP, conventional DDGS, RFHP-DDGS, and LP-DDGS specifically were then calculated by difference as shown below.

TME_n_ for test ingredient (kcal/g) = TME_n_ of ground corn reference diet –
[(TME_n_ of ground corn reference diet – TME_n_ of test diet) / proportion of test ingredient substituted into the corn reference diet]

The kcal/g values were then converted to kcal/kg by multiplying by 1,000.

The standardized AA digestibility of the diets was calculated as described by Engster et al. (1985) and the AA digestibility values of test ingredients were calculated by difference when the test ingredients were fed as a mixture with corn. Basal endogenous AA losses for standardized AA digestibility were determined from AA analysis of excreta collected from cecectomized roosters that were fasted for 48 h. The equations are shown below:

Standardized AA digestibility of diets (%) = [(AA consumed - AA excreted by fed birds + AA excreted by fasted birds) / AA consumed] × 100

where AA consumed (g) = diet intake (g) × AA in diet (%); AA excreted by fed birds (g) = excreta output (g) × AA in excreta (%); AA excreted by fasted birds = excreta output (g) × AA in excreta (%). The AA excreted by fasted birds over a 48 h period were as follows: 49 mg for Asp, 38 mg for Thr, 39 mg for Ser, 80 mg for Glu, 44 mg for Pro, 30 mg for Ala, 24 mg for Cys, 31 mg for Val, 7 mg for Met, 21 mg for Ile, 39 mg for Leu, 22 mg for Tyr, 23 mg for Phe, 28 mg for Lys, 12 mg for His, 31 mg for Arg, and 4 mg for Trp.

The standardized AA digestibility values for CFP, conventional DDGS, RFHP-DDGS, and LP-DDGS were then calculated by difference using the equation:

Standardized AA digestibility of test ingredient (%) = standardized AA digestibility of the ground corn reference diet – [(standardized AA digestibility of the ground corn reference diet - standardized AA digestibility of test ingredient mixture diet with corn) / proportion of test ingredient AA substituted into the mixture diet with corn].

Standardized digestibility values were not evaluated for glycine because of the breakdown of uric acid in excreta to yield glycine during the acid hydrolysis step of AA analysis (Soares et al., 1971).

### Statistical Analysis

The SAS software (SAS Institute; Cary, NC) was used to analyze the data using an ANOVA procedure for a completely randomized design and pairwise treatment comparisons were conducted using the least significant difference test (Carmer and Walker, 1985). Differences in values among treatments were considered significant at *P* < 0.05. Individual roosters were used as the experimental unit for all statistical analyses.

## RESULTS AND DISCUSSION

The analyzed nutrient compositions of CFP and conventional DDGS 1 and 2, obtained from Fluid Quip Technologies, are found in Table 1. With the exception of CFP 3, the CP content of CFP tended to be relatively consistent, ranging from 54.3 to 57.8% on a DM basis. The mean CP content was increased from 31.9% for conventional DDGS1 and 2 to 55.7% for CFP. The CP content of CFP was consistent and slightly higher than the reported CP content of 53% by Corray et al. (2019) (DM basis) for Still Pro, which was produced using a similar process to that used for the CFP samples herein. Corn fermented protein also contained a higher protein content than previously evaluated high protein DDGS produced from the Quick Germ, Quick Germ-Quick Fiber, and Elusieve processes, which were reported to contain 35.9, 49.3, and 40.8% CP (DM basis), respectively. High protein DDGS produced from enzymatic milling resulted in a similar protein content to CFP of 58.5% (DM basis) (Singh et al., 2005; Martinez Amezcua et al., 2007).

**Table 1:** Analyzed composition and TME_n_ of corn fermented protein and conventional distillers dried grains with solubles.^1^

	Corn fermented protein (CFP)									Conventional DDGS^2^			
	1	2	3	4	5	6	7	8	Mean		1	2	Mean
DM (%)	93.6	93.2	92.3	98.3	98.0	93.4	92.4	97.0	94.8		91.9	88.8	90.4
CP (%)	54.8	57.7	50.7	57.8	57.7	54.3	56.5	56.3	55.7		33.6	30.1	31.9
Crude fat (%)	7.1	4.6	5.0	4.2	4.0	4.7	3.5	5.0	4.8		6.4	7.9	7.2
NDF (%)^3^	27.7	29.1	39.7	35.6	32.3	24.1	36.7	37.8	32.9		36.2	43.7	40.0
IDF (%)^3^	26.2	26.5	36.3	25.4	25.1	27.2	30.2	26.2	27.9		39.8	42.9	41.4
SDF (%)^3^	3.5	1.2	2.2	0.9	1.4	4.0	1.2	2.6	2.1		1.4	2.1	1.8
TDF (%)^3^	29.7	27.7	38.5	26.3	26.5	31.2	31.4	28.8	30.0		41.2	45.0	43.1
Ash (%)	5.9	4.7	2.3	4.3	4.1	4.6	3.2	3.1	4.0		6.0	6.5	6.3
Ca (%)	0.05	0.02	0.02	0.02	0.02	0.06	0.03	0.03	0.03		0.03	0.02	0.03
P (%)	1.29	0.78	0.47	0.76	0.77	1.04	0.81	0.82	0.84		1.06	1.25	1.16
Na (%)	0.09	0.08	0.13	0.08	0.08	0.09	0.04	0.04	0.08		0.35	0.31	0.33
Gross energy (kcal/kg)	5,456	5,425	5,346	5,501	5,546	5,384	5,434	5,426	5,440		4,917	5,129	5,023
TME_n_^4^ (kcal/kg)	3,550^ab^	3,553^ab^	3,290^b^	3,767^a^	3,718^a^	3,389	3,684	3,499	3,556		2,832^c^	2,702^c^	2,767
SEM of TME_n_	80	103	43	45	166	50	206	114			25	118	

a-c Values within a row with no common superscript are significantly different (*P* < 0.05). The TME_n_ for CFP6, 7, and 8 were determined in a separate experiment from CFP1, 2, 3, 4, and 5 and conventional DDGS1 and 2; thus, statistical comparisons of CFP6, 7, and 8 were conducted separately from the remaining samples. No significant differences (*P* > 0.05) were observed among TME_n_ values for CFP6, 7, and 8.

1 Products obtained from Fluid Quip Technologies. Values are expressed on a DM basis, excluding DM, which is expressed on an as-fed basis.

2 Conventional DDGS = conventional distillers dried grains with solubles.

3 NDF = neutral detergent fiber; IDF = insoluble dietary fiber; SDF = soluble dietary fiber; TDF = total dietary fiber.

4 TME_n_ values from individually-caged conventional roosters; values are means of 3 roosters for CFP7, 4 roosters for CFP3, CFP6, CFP8 and Conventional DDGS1 and DDGS2, and 6 roosters for CFP1, CFP2, CFP4, and CFP5.

The crude fat content of CFP was found to be generally lower than conventional DDGS (Table 1), with the CFP and conventional DDGS1 and 2 containing a mean of 4.8 and 7.2% crude fat, respectively. The crude fat content of conventional DDGS has been reported to exhibit a wide range of values from 3.2 to 10.1% due to differences in the amount of crude corn oil recovered at ethanol plants (Meloche et al., 2013). Although there were some variations in the crude fat content of CFP, the values generally were approximately 4 to 5%, excluding the higher crude fat content of 7.1% for CFP1.

The NDF content of CFP samples varied and ranged from 24.1 to 39.7%, with a mean of 32.9% (DM basis; Table 1). This was lower than the mean NDF content of 40.0% for conventional DDGS1 and 2, and higher than the sample of Still Pro evaluated by Corray et al. (2019), which contained 24.1% NDF (DM basis). The NDF content of CFP was also found to be higher than DDG produced from enzymatic milling which contained 6.8% NDF, and similar to or lower than DDGS produced from the Elusieve process (Kim et al., 2010). The TDF content of CFP was fairly consistent among samples, excluding CFP3, and contained a mean of 30.0% TDF. The TDF content of CFP and conventional DDGS was primarily composed of IDF, with little SDF being detected. High IDF content compared with SDF in the present study is in good agreement with values reported by Urriola et al. (2010), where conventional DDGS was reported to contain 34% IDF and 1% SDF. The NDF content of CFP samples exceeded the TDF content, excluding CFP1 and CFP6. The cause for the higher NDF content compared with the TDF content may be differences in analytical procedures. The TDF procedure (Method 991.43; AOAC International, 2007) may also allow for more accurate correction of components such as protein, starch, and ash, whereas in the present study the NDF procedure (Method 2002.04; AOAC International, 2007) may not have sufficiently degraded the protein in CFP samples causing the NDF values to be elevated, G. Fahey (University of Illinois, Urbana, IL, personal communication).

The Ca content of all samples was consistently low (Table 1). The mean P content of CFP (0.84%; DM basis) was numerically lower than what was reported for Still Pro by Corray et al. (2019) (1.1%; DM basis). Phosphorus values for conventional DDGS1 and 2 (mean of 1.16%; DM basis) were higher than values from Lumpkins and Batal (2005), Tahir et al. (2012) and the NRC (1994), in which the P content of conventional DDGS was reported to be 0.86, 0.96, and 0.77%, respectively, on a DM basis. Thus, the P content of ethanol co-products may vary among products and samples.

The gross energy of CFP ranged from 5,346 to 5,546 kcal/kg DM, which were higher than values obtained for conventional DDGS1 and 2 (Table 1). For TME_n_ of CFP samples, CFP 3 had a lower (*P* < 0.05) TME_n_ than CFP 4 and 5. The mean TME_n_ for CFP (3,556 kcal/kg DM) was slightly higher than what was reported for Still Pro by Corray et al. (2019) (3,372 kcal/kg) and similar to the TME_n_ of high protein DDG produced from enzymatic milling and the Elusieve pan DDGS (Kim et al., 2010). The TME_n_ of conventional DDGS1 and 2 was 2,832 and 2,702 kcal/kg DM, respectively, which were significantly lower (*P* < 0.05) than CFP1 through 5, and numerically lower than CFP6 through 8. The TME_n_ of conventional DDGS1 and 2 were also lower than what is reported by the NRC (1994) for conventional DDGS. The latter may be due at least partially to the lower fat content of conventional DDGS1 and 2, compared with values reported by the NRC (1994), which is likely the result of recovery of crude corn oil as a separate co-product from the conventional DDGS1 and 2 evaluated in the current study. The effect of oil content on the AME_n_ of DDGS and other corn co-products was previously evaluated by Meloche et al. (2014). In the latter study it was determined that ether extract content had a significant effect on AME_n_, and the addition of ether extract into AME_n_ prediction equations improved the fit of the models.

Analyzed nutrient composition (DM basis) of 2 HP-DDGS samples produced from the back-end fractionation system by Franzenburg is presented in Table 2. High protein-DDGS1 and 2 contained 49.9 and 51.9% CP (DM basis), respectively. These values were substantially higher than conventional DDGS1 and 2 (Table 1) and values reported for conventional DDGS by the NRC (1994), Batal and Dale (2006), Martinez Amezcua et al. (2007), and Urriola et al. (2010), but were similar to DDGS obtained from the Quick Germ-Quick Fiber process (Singh et al., 2005). The crude fat contents of HP-DDGS 1 and 2 were 9.2 and 7.7%, respectively, which are on average slightly higher than conventional DDGS1 and 2 (Table 1), substantially higher than HP-DDG evaluated in the study by Rochell et al. (2011; 3.5%) and slightly lower than the value reported for conventional DDGS by the NRC (1994; 9.7%). The mean NDF and TDF content of HP-DDGS1 and 2 was 34 and 36% (DM basis), respectively. The NDF and TDF content was in good agreement with the 34% NDF value reported for conventional DDGS by the NRC (2012) and 35% TDF value report by Urriola et al. (2010). High protein-DDGS1 and 2 both contained low Ca levels, similar to CFP (Table 1), and contained a P content of 0.69 and 0.73%, respectively. These values are similar, albeit slightly lower, than the value of 0.77% P reported by the NRC (1994) for conventional DDGS.

**Table 2:** Analyzed composition and TME_n_ of high protein-distillers dried grains with solubles.^1^

	HP-DDGS1^2^	HP-DDGS2^2^
DM (%)	93.7	89.5
CP (%)	49.9	51.9
Crude fat (%)	9.2	7.7
NDF (%)^3^	35.9	32.0
IDF (%)^3^	33.7	33.1
SDF (%)^3^	2.1	2.3
TDF (%)^3^	35.9	35.4
Ash (%)	4.1	4.0
Ca (%)	0.01	0.01
P (%)	0.69	0.73
Na (%)	0.01	0.01
Gross energy (kcal/kg)	5,602	5,575
TME_n_^4^ (kcal/kg)	3,307	3,343
SEM of TME_n_	50	75

1 Products obtained from Franzenburg. Values are expressed on a DM basis, excluding DM, which is expressed on an as-fed basis.

2 HP-DDGS = high protein-distillers dried grains with solubles.

3 NDF = neutral detergent fiber; IDF = insoluble dietary fiber; SDF = soluble dietary fiber; TDF = total dietary fiber.

4 TME_n_ values were not significantly different (*P* > 0.05). TME_n_ values are means from 4 individually-caged conventional roosters.

The TME_n_ values of the HP-DDGS were found to be similar (Table 2); values for HP-DDGS1 and 2 were 3,307 and 3,343 kcal/kg DM, respectively. Although TME_n_ of HP-DDGS1 and 2 were higher than conventional DDGS1 and 2 (Table 1), HP-DDGS contained a similar TME_n_ to values reported for conventional DDGS by several groups, which ranged from 3,300 to 3,380 kcal/kg DM (Lumpkins et al., 2004; Kim et al., 2010; NRC 1994).

The analyzed composition (DM basis) of the RFHP-DDGS, LP-DDGS, and conventional DDGS3 and 4, obtained from ST Equipment and Technology LLC fractionation system, are shown in Table 3. The RFHP-DDGS had a CP content of 50.9% and 45.7% (DM basis), respectively (Table 3). The protein content of RFHP-DDGS was increased compared with DDGS from the Quick Germ and Elusieve processes (Singh et al. 2005; Kim et al., 2010) and contained more protein than conventional DDGS1 and 2 (Table 1) and conventional DDGS 3 and 4 (Table 3). The LP-DDGS contained less protein and more fiber than RFHP-DDGS and conventional DDGS3 and 4; this difference resulted from the separation and removal of the RFHP-DDGS fraction, which subsequently yielded a lower protein, higher fiber DDGS in the remaining fraction.

**Table 3:** Analyzed composition and TME_n_ of conventional, reduced fiber high protein, and low protein-distillers dried grains with solubles.^1^

	RFHP- DDGS1^2^	RFHP- DDGS2^2^	LP- DDGS^2^	Conv. DDGS3^2^	Conv. DDGS4^2^
DM (%)	92.5	93.6	92.8	89.4	93.7
CP (%)	50.9	45.7	27.8	33.5	32.6
Crude fat (%)	9.2	11.3	9.7	10.7	5.9
NDF (%)^3^	27.6	21.6	39.9	35.1	35.3
IDF (%)^3^	24.3	19.6	42.1	35.9	36.1
SDF (%)^3^	1.8	1.5	1.6	2.1	2.0
TDF (%)^3^	26.1	21.1	43.8	38.0	38.1
Ash (%)	5.2	4.9	4.8	5.0	5.1
Ca (%)	0.03	0.02	0.02	0.02	0.04
P (%)	1.08	1.01	0.91	1.00	1.01
Na (%)	0.14	0.01	0.01	0.01	0.01
Gross energy (kcal/kg)	5,415	5,559	5,191	5,112	5,292
TME_n_^4^ (kcal/kg)	3,857	3,566^a^	2,968^b^	2,817^b^	3,022^b^
SEM of TME_n_	163	42	183	67	77

a-b Values within a row with no common superscript are significantly different (*P* < 0.05). All samples were evaluated in the same experiment except RFHP-DDGS1.

1 Products obtained from ST Equipment and Technology LLC. Values are expressed on a DM basis, excluding DM, which is expressed on an as-fed basis.

2 RFHP-DDGS = reduced fiber high protein-distillers dried grains with solubles; LP-DDGS = low protein-distillers dried grains with solubles; Conv. DDGS = conventional distillers dried grains with solubles.

3 NDF = neutral detergent fiber; IDF = insoluble dietary fiber; SDF = soluble dietary fiber; TDF = total dietary fiber.

4 TME_n_ values are means from 5 individually-caged conventional roosters for Conv. DDGS3 and 4, RFHP-DDGS2, and LP-DDGS, and 6 individually-caged roosters for RFHP-DDGS1.

Crude fat content was relatively consistent among RFHP-DDGS1, 2, and LP-DDGS (Table 3), ranging from 9.2 to 11.3% (DM basis); however, the crude fat content of conventional DDGS3 and 4 was inconsistent, being 10.7 and 5.9%, respectively. This variation may be caused by differences in the amount of crude corn oil recovered from thin stillage (Meloche et al., 2013). In contrast to the variability observed for crude fat content, Ca and P concentrations were consistent among all samples in Table 3, with Ca content being low and P content ranging from 0.9 to 1.1% (DM basis).

The TME_n_ of RFHP-DDGS1 and 2 were 3,857 and 3,566 kcal/kg DM, respectively, and the TME_n_ of RFHP-DDGS2 was higher (*P* < 0.05) than LP-DDGS and conventional DDGS3 and 4 (Table 3). The higher TME_n_ of the RFHP-DDGS2 was partially due to the higher protein and lower fiber content compared with LP-DDGS and conventional DDGS3 and 4. Conventional DDGS3 contained the lowest TME_n_, although it did not differ (*P* > 0.05) from conventional DDGS 4 and LP-DDGS.

Total AA concentrations, standardized AA digestibility values, and digestible AA concentrations of CFP and conventional DDGS1 and 2 are presented in Tables 4 through 7. All CFP samples contained higher concentrations of all AA compared with conventional DDGS (Table 4) and had a higher total Lys to CP ratio of 3.66 vs. 3.01, respectively (data not shown), indicating a higher protein quantity and quality for CFP. The mean standardized AA digestibility of CFP ranged from 89 to 94% among samples (Tables 5 and 6). There were generally no consistent differences in AA digestibility values for the CFP samples compared with conventional DDGS, except for lower (*P* < 0.05) Trp digestibility values for the conventional DDGS samples (Table 5). The standardized AA digestibility values for CFP are in good agreement with the digestibility values reported by Corray et al. (2019) for Still Pro, a product similar to CFP, which ranged from 84 to 94% among individual AA. The AA digestibility of CFP samples was also found to be relatively consistent among samples, with few differences (*P* < 0.05) being observed aside from the generally higher digestibility values of CFP3 relative to other CFP samples (Table 5). Mean digestibility of AA for the other 7 CFP samples varied from 89 to 91% (Tables 5 and 6).

**Table 4:** Total amino acid concentrations in corn fermented protein and conventional distillers dried grains with solubles (%, DM basis).^1^

Amino acid	Corn fermented protein								Conv. DDGS^2^	
	1	2	3	4	5	6	7	8	1	2
Asp	3.76	3.80	3.51	3.79	3.78	3.95	4.09	4.05	1.97	1.78
Thr	2.15	2.18	1.96	2.16	2.15	2.22	2.29	2.25	1.16	1.08
Ser	2.36	2.36	2.08	2.33	2.33	2.41	2.49	2.38	1.33	1.19
Glu	8.58	9.11	8.11	9.07	9.01	8.61	8.91	8.85	4.99	3.96
Pro	4.52	4.76	4.11	4.74	4.78	3.96	4.10	4.13	2.72	2.20
Gly	2.09	2.11	1.92	2.13	2.10	2.10	2.16	2.18	1.23	1.13
Ala	3.88	4.13	3.58	4.12	4.09	3.88	3.99	3.98	2.34	1.98
Cys	1.08	1.17	1.01	1.17	1.16	1.07	1.14	1.13	0.67	0.62
Val	3.09	3.14	2.84	3.17	3.16	2.98	3.08	3.11	1.62	1.48
Met	1.14	1.27	1.14	1.25	1.24	1.18	1.23	1.22	0.66	0.57
Ile	2.38	2.47	2.21	2.48	2.48	2.36	2.45	2.45	1.24	1.11
Leu	6.58	7.14	6.07	7.10	7.08	6.52	6.75	6.69	3.86	3.25
Tyr	2.19	2.35	1.85	2.25	2.27	2.43	2.48	2.45	1.13	0.97
Phe	2.92	3.10	2.69	3.08	3.08	2.91	3.00	2.98	1.63	1.41
Lys	2.12	1.91	1.81	1.84	1.84	2.18	2.33	2.31	0.96	0.96
His	1.54	1.51	1.38	1.52	1.51	1.48	1.55	1.56	0.88	0.80
Arg	2.53	2.51	2.21	2.49	2.49	2.55	2.63	2.64	1.40	1.31
Trp	0.49	0.53	0.49	0.49	0.49	0.55	0.55	0.56	0.25	0.23

1 Products obtained from Fluid Quip Technologies.

2 Conv. DDGS = conventional distillers dried grains with solubles.

**Table 5:** Standardized digestibility values of amino acids in corn fermented protein and conventional distillers dried grains with solubles (%).^1^

	CFP1^2^		CFP2^2^		CFP3^2^		CFP4^2^		CFP5^2^		Conv. DDGS1^2^		Conv. DDGS2^2^	
Amino acid	Digest. value^3^	SEM	Digest. value^3^	SEM	Digest. value^3^	SEM	Digest. value^3^	SEM	Digest. value^3^	SEM	Digest. value^3^	SEM	Digest.value^3^	SEM
Asp	88^ab^	1.1	88^ab^	1.3	91^a^	0.8	86^bc^	1.4	85^bc^	1.3	85^bc^	2.9	82^c^	1.4
Thr	86^ab^	1.5	87^ab^	1.6	90^a^	1.2	86^ab^	1.6	84^b^	1.1	85^ab^	2.7	82^b^	1.4
Ser	89^a^	1.7	90^a^	1.9	93^a^	1.0	89^a^	1.3	88^a^	1.1	91^a^	3.5	89^a^	1.6
Glu	93^b^	0.7	94^ab^	0.6	96^a^	0.2	93^b^	0.8	92^b^	0.7	93^a^	1.7	92^b^	1.5
Pro	92^b^	0.9	92^b^	1.1	95^a^	0.2	92^b^	1.0	91^b^	0.7	91^b^	1.5	89^b^	1.3
Ala	92^b^	0.8	93^ab^	0.6	95^a^	0.3	92^b^	0.9	91^b^	0.8	92^ab^	1.8	90^b^	1.4
Cys	85^b^	2.2	86^ab^	2.2	92^a^	1.1	86^ab^	1.9	85^b^	2.1	84^b^	4.4	81^b^	1.9
Val	89^b^	1.1	90^b^	1.1	95^a^	0.2	89^b^	1.2	88^b^	0.8	91^ab^	2.8	90^b^	1.9
Met	91^b^	0.4	93^b^	0.5	97^a^	0.1	92^b^	0.9	92^b^	0.5	94^ab^	2.0	94^ab^	2.4
Ile	91^ab^	0.8	92^a^	0.7	93^a^	0.2	91^ab^	1.0	90abc	0.9	88^bc^	2.8	87^c^	1.9
Leu	94^b^	0.5	95^ab^	0.4	97^a^	0.1	94^b^	0.6	94^b^	0.6	95^ab^	1.6	94^b^	1.1
Tyr	93^b^	0.9	95^ab^	0.6	97^a^	0.2	93^b^	1.0	93^b^	0.7	93^b^	2.3	92^b^	1.3
Phe	93^b^	0.7	93^ab^	0.6	96^a^	0.1	93^b^	0.8	92^b^	0.6	92^b^	2.3	91^b^	1.4
Lys	83^b^	1.5	83^b^	1.1	91^a^	0.8	81^b^	2.2	80^b^	1.6	83^b^	5.0	82^b^	3.2
His	90^ab^	1.2	91^ab^	0.8	93^a^	0.3	89^bc^	1.2	87^c^	1.1	90abc	1.5	88^bc^	0.7
Arg	93^b^	1.0	95^ab^	0.8	98^a^	0.5	93^b^	0.9	94^b^	0.9	94^b^	3.0	93^b^	1.6
Trp	95^a^	0.9	95^a^	0.8	94^a^	0.7	93^a^	1.2	93^a^	0.5	84^b^	2.2	78^c^	0.9
Mean	90		91		94		90		89		90		88	

a-d Standardized digestibility values within a row with no common superscript are significantly different (*P* < 0.05).

1 Products obtained from Fluid Quip Technologies.

2 CFP = corn fermented protein; Conv. DDGS = conventional distillers dried grains with solubles.

3 Values are means of 3 individually-caged cecectomized roosters for Conv. DDGS2, 4 roosters for CFP3 and Conv. DDGS1, and 6 roosters for CFP1, CFP2, CFP4, and CFP5.

**Table 6:** Standardized digestibility values of amino acids in corn fermented protein (%).^1^

Amino acid	CFP6^2^		CFP7^2^		CFP8^2^	
	Digest value^3^	SEM	Digest value^3^	SEM	Digest value^3^	SEM
Asp	86	1.9	87	0.6	88	2.0
Thr	86	2.6	87	1.0	87	2.3
Ser	87	2.8	89	0.9	88	1.1
Glu	91	1.7	93	0.5	93	1.3
Pro	89	1.9	91	0.7	90	1.0
Ala	90	1.6	92	0.4	92	0.8
Cys	79	3.7	81	1.5	79	2.6
Val	89	2.0	91	0.5	90	0.7
Met	93	1.7	94	0.4	94	0.7
Ile	87	2.0	89	0.4	89	1.0
Leu	93	1.4	95	0.3	94	0.4
Tyr	91	1.8	92	0.6	92	1.1
Phe	91	1.7	93	0.3	92	0.6
Lys	86	2.4	89	0.9	89	2.4
His	90	1.8	90	0.6	91	2.2
Arg	92	2.3	94	0.5	92	0.6
Trp	90	1.7	91	1.1	90	1.5
Mean	89		90		90	

1 Products obtained from Fluid Quip Technologies. Standardized AA digestibility values within rows were not significantly different (*P* > 0.05).

2 CFP = corn fermented protein.

3 Values are means of 3 individually-caged cecectomized roosters for CFP8, and 4 roosters for CFP6 and CFP7.

The standardized AA digestibility of conventional DDGS1 and 2 (Table 5) were generally higher than values reported for conventional DDGS (Batal and Dale, 2006; Kim et al., 2008, 2010). In particular, Lys digestibility was reported to be 70% by Batal and Dale (2006) and 74% by Kim et al. (2008), with 1 of the samples evaluated in the former study having a Lys digestibility of only 46% when using the same cecectomized rooster assay as that used herein. The average Lys digestibility of conventional DDGS in the current study was approximately 83%. Higher Lys digestibility values observed in the present study may be due to product manufacturing with the use of milder drying processes, such as ring dryers, which reduce the occurrence of Maillard reactions (Moughan et al., 1999). Accompanied by elevated AA concentrations compared with conventional DDGS and high AA digestibility values, CFP contained much higher digestible concentrations of each AA compared with conventional DDGS (Table 7). For example, the mean digestible concentrations of Lys, Met+Cys, and Thr were increased from 0.79, 1.12, and 0.94% in conventional DDGS, respectively, to 1.74, 2.06, and 1.88% in CFP, respectively. The increase in digestible Lys in CFP can likely be partially attributed to the increased concentration of yeast in CFP compared with conventional DDGS; CFP is estimated to contain 25% yeast (DM basis) P. Williams (Fluid Quip Technologies, Cedar Rapids, IA, personal communication). Yeast have been found to contain an improved AA balance compared with corn, as they contain approximately 5.5% total Lys as a % of CP compared with 2.7% for corn (Belyea et al., 2004).

**Table 7:** Digestible concentrations of amino acids in corn fermented protein and conventional distillers dried grains with solubles (%, DM basis).^1^

Amino acid	Corn fermented protein									Conv. DDGS^2^		
	1	2	3	4	5	6	7	8	Mean	1	2	Mean
Asp	3.30	3.34	3.21	3.26	3.19	3.38	3.56	3.56	3.35	1.68	1.46	1.57
Thr	1.85	1.90	1.77	1.85	1.80	1.90	2.00	1.96	1.88	0.99	0.89	0.94
Ser	2.08	2.11	1.94	2.08	2.05	2.09	2.22	2.09	2.08	1.20	1.06	1.13
Glu	7.98	8.54	7.80	8.45	8.31	7.83	8.27	8.22	8.18	4.67	3.63	4.15
Pro	4.18	4.40	3.92	4.36	4.34	3.51	3.73	3.71	4.02	2.46	1.96	2.21
Ala	3.55	3.83	3.38	3.78	3.72	3.49	3.66	3.65	3.63	2.15	1.79	1.97
Cys	0.91	1.01	0.92	1.00	0.98	0.85	0.92	0.90	0.94	0.57	0.50	0.54
Val	2.74	2.82	2.71	2.82	2.77	2.64	2.80	2.80	2.76	1.48	1.33	1.41
Met	1.05	1.18	1.11	1.15	1.14	1.09	1.16	1.14	1.13	0.63	0.54	0.59
Ile	2.16	2.26	2.06	2.25	2.22	2.06	2.19	2.19	2.17	1.10	0.97	1.04
Leu	6.21	6.78	5.89	6.70	6.65	6.05	6.40	6.29	6.37	3.67	3.06	3.37
Tyr	2.04	2.22	1.80	2.09	2.10	2.21	2.28	2.26	2.13	1.06	0.89	0.98
Phe	2.70	2.90	2.58	2.85	2.85	2.63	2.78	2.74	2.75	1.50	1.27	1.39
Lys	1.76	1.59	1.64	1.49	1.47	1.86	2.07	2.06	1.74	0.79	0.79	0.79
His	1.39	1.37	1.28	1.36	1.32	1.33	1.40	1.42	1.36	0.78	0.70	0.74
Arg	2.36	2.37	2.17	2.32	2.33	2.33	2.46	2.43	2.35	1.32	1.22	1.27
Trp	0.47	0.49	0.46	0.46	0.45	0.49	0.50	0.51	0.48	0.21	0.18	0.20

1 Products obtained from Fluid Quip Technologies. Digestible concentration = (total x standardized digestibility value)/100.

2 Conv. DDGS = conventional distillers dried grains with solubles.

Total AA concentrations of HP-DDGS2 were found to be slightly higher than HP-DDGS1 (Table 8). Both samples contained numerically increased concentrations of AA compared with conventional DDGS1 and 2 (Table 4). Concentrations of AA were also higher than values reported for conventional DDGS by the NRC (1994). For example, HP-DDGS1 and 2 contained 1.7% and 1.8% total Lys, respectively, compared with 0.8% in conventional DDGS (DM basis; NRC, 1994). The standardized AA digestibility values for the 2 HP-DDGS did not differ significantly (*P* > 0.05) between samples for most AA, and HP-DDGS contained a mean digestibility of 90% for all AA combined. Digestibility values for HP-DDGS were similar for most AA compared with values determined in cecectomized roosters reported by Kim et al. (2008) for a higher protein DDG containing 48% CP (DM basis), and the mean AA digestibility of HP-DDGS1 was numerically higher than conventional DDGS1 and 2 in the present study. The largest difference among standardized AA digestibility values for HP-DDGS and values reported for high protein DDG by Kim et al. (2008) were for Lys digestibility, where Lys digestibility was found to be substantially lower for high protein DDG in the Kim et al. (2008) study. Reduced Lys digestibility values for DDGS in previous studies may be attributed to the extensive drying that is required to produce DDGS, which can cause Maillard reactions to occur between reducing sugars and the free ε-NH2 group in the side chain of Lys (Kwiatkowski et al., 2006). Similar to total AA concentrations, digestible AA concentrations were also numerically higher for HP-DDGS compared with conventional DDGS1 and 2 (Table 7). For example, the digestible concentrations of Lys, Met+Cys, and Thr were 1.50%, 1.79%, and 1.60% for HP-DDGS1, and 1.56%, 1.75%, and 1.60% for HP-DDGS2.

**Table 8:** Total concentrations, standardized digestibility values, and digestible concentrations of amino acids in high protein-distillers dried grains with solubles (%, DM basis).^1^

Amino acid	HP-DDGS1^2^				HP-DDGS2^2^			
	Total	Digest. value^3^	SEM^4^	Digest. conc.^5^	Total	Digest. value^3^	SEM^4^	Digest. conc.^5^
Asp	3.19	87^a^	1.0	2.77	3.32	83^b^	0.7	2.74
Thr	1.81	88^a^	1.5	1.60	1.90	84^a^	1.0	1.60
Ser	2.01	90^a^	1.2	1.81	2.08	86^b^	1.0	1.79
Glu	7.39	94^a^	0.6	6.91	7.59	91^b^	0.7	6.91
Pro	3.81	92^a^	1.1	3.52	3.89	90^a^	0.7	3.48
Gly	1.77	-	-	-	1.84	-	-	-
Ala	3.47	93^a^	0.8	3.22	3.55	91^a^	0.8	3.23
Cys	0.95	85^a^	1.7	0.81	0.96	79^b^	1.4	0.76
Val	2.64	92^a^	0.9	2.42	2.75	90^a^	0.9	2.46
Met	1.04	95^a^	0.4	0.98	1.06	93^a^	0.8	0.99
Ile	2.09	90^a^	1.0	1.89	2.19	89^a^	0.9	1.94
Leu	6.02	95^a^	0.6	5.69	6.19	93^a^	0.7	5.76
Tyr	1.92	93^a^	1.2	1.78	2.01	91^a^	1.3	1.82
Phe	2.60	93^a^	0.7	2.43	2.70	91^a^	0.8	2.47
Lys	1.70	88^a^	1.6	1.50	1.83	86^a^	1.7	1.56
His	1.32	90^a^	1.1	1.19	1.40	88^a^	0.7	1.23
Arg	2.09	94^a^	0.5	1.97	2.18	92^a^	0.9	2.00
Trp	0.30	87^a^	1.8	0.26	0.34	82^a^	0.7	0.28
Mean		91				88		

a-b Standardized digestibility values within the same row with no common superscript are significantly different (*P* < 0.05).

1 Products obtained from Franzenburg.

2 HP-DDGS = high protein distillers dried grains with solubles.

3 Values are means of 4 individually-caged cecectomized roosters.

4 SEM for standardized digestibility values.

5 Digestible concentration = (total x standardized digestibility value)/100.

Total AA concentrations of RFHP-DDGS were higher than concentrations for LP-DDGS and conventional DDGS3 and 4 (Table 9). The RFHP-DDGS1 contained greater total concentrations of AA than RFHP-DDGS2, which was expected based on the higher CP content of RFHP-DDGS1, shown in Table 3. The mean AA digestibility for the 2 RFHP-DDGS, LP-DDGS, and the 2 conventional DDGS were 90, 86, and 87%, respectively (Table 10). The digestibility of Lys exhibited a large numerical difference between RFHP-DDGS samples. As discussed earlier, the numerically higher digestibility of Lys in RFHP-DDGS2 may have been largely due to utilization of a milder drying process compared with RFHP-DDGS1. Standardized AA digestibility values for RFHP-DDGS2, LP-DDGS, and conventional DDGS3 and 4 exhibited few differences (*P* < 0.05). Digestibility values from Table 10 were also comparable with cecectomized rooster values reported for conventional DDGS and modified higher protein DDG and DDGS by Kim et al. (2010). Digestible AA concentrations of the 2 RFHP-DDGS samples were substantially higher than LP-DDGS and conventional DDGS3 and 4 (Table 11). For example, the mean digestible concentrations of Lys, Met, Cys, and Thr in RFHP-DDGS1 and 2 were 1.00, 0.82, 0.76, and 1.44%, respectively. These values are higher compared with conventional DDGS3 and 4 which contained a mean of 0.82, 0.53, 0.48, and 0.95% digestible Lys, Met, Cys, and Thr, respectively. Conventional DDGS3 and 4 contained similar digestible AA concentrations to conventional DDGS1 and 2 (Table 7), while LP-DDGS tended to contain slightly lower digestible AA concentrations compared with all evaluated conventional DDGS samples.

**Table 9:** Total concentrations of amino acids in reduced fiber high protein, low protein, and conventional distillers dried grains with solubles (%, DM basis).^1^

Amino acid	RFHP-DDGS1^2^	RFHP-DDGS2^2^	LP-DDGS^2^	Conv. DDGS3^2^	Conv. DDGS4^2^
Asp	3.06	2.76	1.63	2.04	1.95
Thr	1.79	1.61	1.00	1.17	1.16
Ser	2.22	2.00	1.21	1.44	1.43
Glu	8.01	7.03	3.62	4.94	4.55
Pro	4.17	3.83	2.14	2.60	2.59
Gly	1.65	1.72	1.14	1.34	1.30
Ala	3.60	3.35	1.86	2.33	2.26
Cys	0.96	0.85	0.49	0.63	0.60
Val	2.45	2.37	1.36	1.61	1.61
Met	0.96	0.85	0.45	0.58	0.57
Ile	2.01	1.95	1.09	1.26	1.30
Leu	6.45	5.84	3.01	3.66	3.75
Tyr	2.12	1.76	0.96	1.17	1.17
Phe	2.89	2.52	1.44	1.68	1.73
Lys	1.38	1.27	0.90	1.06	1.02
His	1.29	1.19	0.73	0.87	0.85
Arg	2.06	1.90	1.22	1.48	1.41
Trp	0.34	0.26	0.18	0.19	0.21

1 Products obtained from ST Equipment and Technology LLC.

2 RFHP-DDGS = reduced fiber high protein-distillers dried grains with solubles; LP-DDGS = low protein-distillers dried grains with solubles; Conv. DDGS = conventional distillers dried grains with solubles.

**Table 10:** Standardized digestibility values of amino acids in reduced fiber high protein, low protein, and conventional distillers dried grains with solubles (%, DM basis).^1^

Amino acid	RFHP-DDGS1^2^		RFHP-DDGS2^2^		LP-DDGS^2^		Conv. DDGS3^2^		Conv. DDGS4^2^	
	Digest. value^3^	SEM	Digest. value^3^	SEM	Digest. value^3^	SEM	Digest. value^3^	SEM	Digest. value^3^	SEM
Asp	77	3.4	85^a^	2.7	77^b^	2.2	79^ab^	2.8	83^ab^	2.3
Thr	85	1.1	85^a^	3.0	78^a^	2.5	78^a^	3.5	83^a^	3.0
Ser	89	1.5	92^a^	2.6	88^a^	2.8	87^a^	3.1	91^a^	1.9
Glu	92	0.9	93^a^	1.7	89^a^	1.5	90^a^	2.0	92^a^	1.1
Pro	92	0.8	92^a^	1.9	86^b^	1.5	88^ab^	2.2	89^ab^	1.8
Ala	90	1.0	93^a^	1.5	89^ab^	1.2	88^b^	2.1	91^ab^	1.2
Cys	84	1.8	83^a^	4.4	75^a^	4.5	75^a^	6.4	82^a^	4.3
Val	90	1.2	93^a^	1.9	89^a^	1.9	87^a^	3.2	91^a^	1.8
Met	89	1.2	95^a^	1.4	92^a^	1.1	91^a^	2.0	93^a^	1.3
Ile	89	1.3	91^a^	1.7	86^ab^	1.4	84^b^	2.8	89^ab^	1.6
Leu	95	0.6	96^a^	1.0	93^a^	1.0	92^a^	1.8	95^a^	0.9
Tyr	93	1.2	95^a^	1.6	92^a^	1.4	90^a^	2.2	94^a^	1.2
Phe	92	1.0	94^a^	1.4	90^ab^	1.1	89^b^	2.4	93^ab^	1.2
Lys	68	4.3	84^a^	3.4	78^ab^	1.8	75^b^	3.9	81^ab^	2.4
His	84	0.9	90^a^	1.6	84^b^	1.4	85^ab^	1.9	88^ab^	1.6
Arg	91	1.8	95^a^	1.8	93^a^	1.8	91^a^	2.7	94^a^	1.2
Trp	92	0.9	88^a^	1.6	81^a^	3.3	68^b^	3.3	88^a^	3.6
Mean	88		91		86		85		89	

a-b Standardized digestibility values within the same row with no common superscript are significantly different (*P* < 0.05).

1 Products obtained from ST Equipment and Technology LLC.

2 RFHP-DDGS = reduced fiber high protein-distillers dried grains with solubles; LP-DDGS = low protein-distillers dried grains with solubles; Conv. DDGS = conventional distillers dried grains with solubles.

3 Values are means of 5 individually-caged cecectomized roosters for Conv. DDGS3 and 4, RFHP-DDGS2, and LP-DDGS and 6 individually-caged roosters for RFHP-DDGS1. All samples were evaluated in the same experiment excluding RFHP-DDGS1.

**Table 11:** Digestible amino acid concentrations in reduced fiber high protein, low protein, and conventional distillers dried grains with solubles (%, DM basis).^1^

Amino acid	RFHP-DDGS1^2^	RFHP-DDGS2^2^	LP-DDGS^2^	Conv. DDGS3^2^	Conv. DDGS4^2^
Asp	2.36	2.34	1.26	1.60	1.62
Thr	1.52	1.37	0.78	0.92	0.97
Ser	1.98	1.84	1.07	1.25	1.30
Glu	7.33	6.50	3.22	4.44	4.16
Pro	3.85	3.53	1.83	2.27	2.31
Ala	3.25	3.12	1.65	2.06	2.07
Cys	0.81	0.71	0.37	0.47	0.49
Val	2.21	2.20	1.20	1.39	1.47
Met	0.85	0.80	0.41	0.53	0.53
Ile	1.78	1.78	0.93	1.06	1.15
Leu	6.10	5.58	2.80	3.37	3.54
Tyr	1.97	1.66	0.88	1.06	1.10
Phe	2.66	2.37	1.30	1.49	1.60
Lys	0.94	1.07	0.70	0.80	0.83
His	1.08	1.06	0.61	0.74	0.75
Arg	1.88	1.80	1.13	1.34	1.33
Trp	0.31	0.23	0.15	0.13	0.19

1 Products obtained from ST Equipment and Technology LLC. Digestible concentration = (total x standardized digestibility value)/100.

2 RFHP-DDGS = reduced fiber high protein-distillers dried grains with solubles; LP-DDGS = low protein-distillers dried grains with solubles; Conv. DDGS = conventional distillers dried grains with solubles

In summary, the TME_n_ values for the increased protein ethanol co-products CFP, HP-DDGS, and RFHP- DDGS were substantially higher than for conventional DDGS. The AA digestibility of the increased protein co-products was generally similar to conventional DDGS, but the digestible AA concentrations were higher than conventional DDGS for all AA. The results from this study indicate that the energy and protein in the increased protein co-products produced from back-end fractionation of corn DDGS are well digested and utilized by poultry and they contain increased nutritional value compared with conventional DDGS.

## DISCLOSURES

The authors declare that they have no known competing financial interests or personal relationships that could have appeared to influence the work reported in this paper.
